# Images with hidden information data set for information retrieval usage

**DOI:** 10.1016/j.dib.2019.104397

**Published:** 2019-08-16

**Authors:** Peter Pangestu, Dennis Gunawan, Seng Hansun

**Affiliations:** Informatics Department, Universitas Multimedia Nusantara, Tangerang, Indonesia

**Keywords:** Hidden messages, Histogram equalization, Images, Information retrieval, OCR

## Abstract

The main task in Optical Character Recognition (OCR) is to get and convert all the text characters on an image as a plain text data. However, if the image has low contrast and low exposure, an issue may occur. The characters may be hidden and can't be recovered completely. One solution that has been done and reported in 2017 is by applying histogram equalization as a pre-processing step in OCR. Here, we deliver a total of 30 sample data, some of which had been used on the research's experiment reported in 2017, and some others were added later.

**Table:** Specifications table

Subject area	*Computer Science*
More specific subject area	*Information retrieval*
Type of data	*Table, image*
How data was acquired	*Data set was collected and created using publicly available data sets, taken from Google images.*
Data format	*Raw and Filtered*
Experimental factors	*All images that have been collected were images with a dark background and low contrast*
Experimental features	*On each collected image, some texts were added. The text color selection was made by adding a hexadecimal value of 10 for each RGB component of the background image*
Data source location	*Google Images database*
Data accessibility	*The data are available with this article.*
Related research article	*Pangestu P., Gunawan D., Hansun S. Histogram Equalization Implementation in the Preprocessing Phase on Optical Character Recognition. International Journal of Technology (IJTech), Vol.8, No.5, pp.947–956. 2017.*

**Table:** 

**Value of the data** • This data was collected from various resources, which then filtered and modified by adding some texts, that can be used by other researchers as a benchmark data set in information retrieval field • The texts inserted in the images follow some rules that allow being defined and modified by other researchers in their respective fields • For future investigations, the Histogram Equalization method employed in this study can be further extended to enhance the image's contrast on the data set

## 1 Data

All the data being shared here are images that had been collected from Google Images database [1]. A total of 30 images have been successfully collected. All the images have a common pattern, i.e., they all have a dark background, low contrast, and are not too crowded. The image analysis aimed to determine if the image conditions were in accordance with the application of histogram equalization in general.

**Fig. 1:**
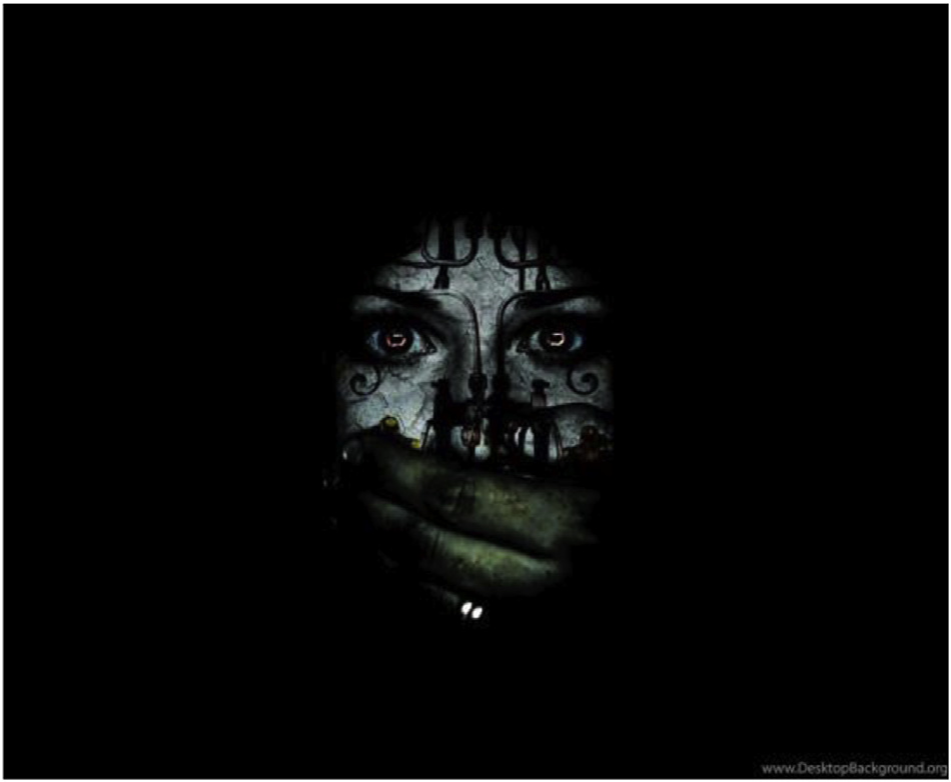
The original image.

**Fig. 2:**
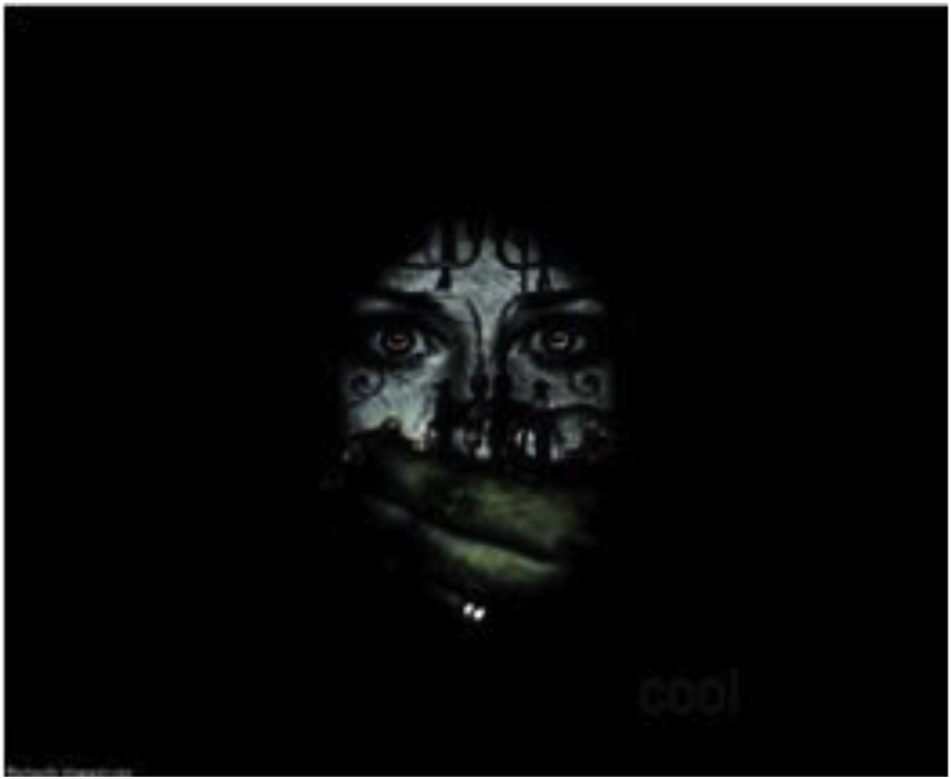
The filtered and modified image. It contains a ‘cool’ word.

All the image data in this article were filtered and modified by adding some texts. Fig. 1 describes the original image which was taken from www.desktopbackground.org. The figure then was filtered and cropped to make sure that it didn't have any other texts visible to the user. Next, we added some texts that follow some rules so that it can't be easily read by the user. Fig. 2 shows the filtered and modified image that has been added with a ‘cool’ word.

## 2 Experimental design, materials, and methods

To collect and generate the data set needed, all the images that have been collected from Google images database were added some texts. To make sure that the added texts were not easily read, a condition was given for the text colorization. Selection of the text color was done by adding a hexadecimal value of 10 for each RGB components of the background. For example, RGB #000000 become RGB #101010. Table 1 shows the data set that has been built for the experimental purpose in Pangestu et al. [2] research and some others that had been added later after the publication of the research paper.

**Table 1:** Data set built.

No	Image's Caption	Added Texts	Resulted Image
1	sugar.png (https://www.pinterest.com/pin/574209021233308540)	lele jumbo	
2	manus.png (http://darksouls.wdfiles.com/local--files/bosses/manus-father-of-the-abyss-large.jpg)	I WILL KILL YOU	
3	phantom-assassin-dota-2-dota-2740 (1).png (https://www.tokkoro.com/1991615-dota-2.html)	assasin	
4	cool_dark_wallpaper.png (https://www.desktopbackground.org/wallpaper/wide-hd-scary-wallpapers-640678)	cool	
5	12June2012-Low-light-focusing-lrg.png (https://www.sabisabi.com/photography/low-light-focusing/)	LION KING	
6	images (5).jpg	the dragon	
7	images.jpg (https://www.pinterest.com/pin/277886239483458855)	THERE’S LIGHT EVEN IN THE DARKEST PLACES.	
8	vvv.png (https://memerino.me/v-for-vendetta)	V FOR VENDETA	
9	dw1.png (https://www.wallpaperflare.com/photography-black-man-hat-white-shirt-red-tie-dark-background-wallpaper-mhrzm)	anonymous	
10	hitman-suit-dark-hd-wallpaper-vvallpaper-net-by-aimanchong-dark-wallpaper-hd.JPG (https://www.deviantart.com/aimanchong/art/Hitman-Suit-Dark-HD-Wallpaper-Vvallpaper-Net-403217315)	WITH A TIE	
11	1003738-dark.png (https://wallpapercave.com/w/hMJvzuy)	HMMMM	
12	lambhorgini-dark- wallpaper-1366 × 768.png (http://wallpaperswide.com/lamborghini_dark-wallpapers.html)	lambhorgini	
13	glows_in_the_dark-wide.png (http://wallpaperswide.com/glows_in_the_dark_fwa_nordic-wallpapers.html)	GLOW	
14	41164.png (https://wall.alphacoders.com/big.php?i=41164)	vampire	
15	nod32_robot_black_white_26279_1920 × 1080.png (http://www.pyscorp.com/wp-content/uploads/2015/09/nod32_robot_black_white_26279_1920x1080.jpg)	ROBOT	
16	Valind-and-Rogue-Flash-Bender-2.jpg (https://rogueflash.com/blogs/blog/47329157-low-key-film-noir-portrait-with-erik-valind-and-flashbender-2)	BEAUTIFUL	
17	low-key_cat.jpg (https://fr.wikipedia.org/wiki/Low-key_lighting#/media/Fichier:Low-key_cat.jpg)	Low light cat	
18	7d0GbFV.png (source: https://www.desktopbackground.org/wallpaper/tegami-bachi-wiki-wikia-470514)	ON YOUR MIND	
19	610.png (https://wpmisc.com/wallpaper-439592#)	DOGGY	
20	20131108-_a4q8743-edit-edit-2.jpg (https://photowestguy.files.wordpress.com/2014/05/20131108-_a4q8743-edit-edit-2.jpg)	FLOWER	
21	black_hat.jpg	A MAN WITH BLACK HAT	
22	6808332673_3fe8de3766_b.jpg (https://www.flickr.com/photos/graham_scarborough/6808332673)	yellow flower	
23	Black_and_white_photography_tips_DCM120.feature.darktones_lowkey.jpg (https://deconstructionchimera.wordpress.com/2013/11/01/light-on-the-face/)	this is low light photo	
24	dark wallpaper 28.png (https://www.google.co.id/imgres?imgurl=https://pbs.twimg.com/profile_images/1101929530112049152/lPq23KHj_400x400.png&imgrefurl=https://twitter.com/zehra_jimin&h=400&w=400&tbnid=llPfDiMnPw9UdM&tbnh=225&tbnw=225&usg=K_zR9lfcIlXxY73cSRODsYahmhnC0=&hl=en-ID&docid=LxlP518dIfAfEM)	DARK WALLPAPER	
25	dark_wallpaper_a3222.jpg (https://www.desktopbackground.org/wallpaper/dark-wallpapers-hd-wallpaper-download-xpx-hd-wallpaper-dark-wallpapers-for-mobile-1080p-android-laptop-windows-7-mac-1366x768-iphone-nature-jpg-202077)	kucing	
26	final_500.png (https://ricknunn.com/strobist-setups/how-i-shoot-lowkey-portrait)	FINAL 500	
27	jf93UfN.png (https://wallpaper-gallery.net/single/wallpaper-of-black-13.html)	PARDON MY FACE	
28	download (5).jpg	your face	
29	dark_souls_wallpaper_a3243.png (http://wallpapersafari.com/w/n3ZANU)	CAT FACE	
30	sony-vaio-wallpaper-black-wallpaper-hd-10c.png (http://flowermoundlocal.info/fond-ecran-hd-iphone/fond-ecran-hd-iphone-beautiful-unique-iphone-7-wallpaper-hd-original/)	NEED MOTIVATION	
